# Restructuring and Hydrogen Evolution on Pt Nanoparticle†
†Electronic supplementary information (ESI) available: Discussions on the structures of Pt clusters and the stability of the subsurface H atoms in Pt cluster, TS structure of H–H coupling on {111} facets of Pt_44_H_80_, *XYZ* coordinate of Pt_44_ and Pt_44_H_80_. Movie of structure evolution at Pt_44_H_50_ See DOI: 10.1039/c4sc02806f



**DOI:** 10.1039/c4sc02806f

**Published:** 2014-11-26

**Authors:** Guang-Feng Wei, Zhi-Pan Liu

**Affiliations:** a Shanghai Key Laboratory of Molecular Catalysis and Innovative Materials , Department of Chemistry , Key Laboratory of Computational Physical Science (Ministry of Education) , Fudan University , Shanghai 200433 , China . Email: zpliu@fudan.edu.cn

## Abstract

The restructuring of nanoparticles at the *in situ* condition is a common but complex phenomenon in nanoscience. Here, we present the first systematic survey on the structure dynamics and its catalytic consequence for hydrogen evolution reaction (HER) on Pt nanoparticles, as represented by a magic number Pt_44_ octahedron (∼1 nm size). Using a first principles calculation based global structure search method, we stepwise follow the significant nanoparticle restructuring under HER conditions as driven by thermodynamics to expose {100} facets, and reveal the consequent large activity enhancement due to the marked increase of the concentration of the active site, being identified to be apex atoms. The enhanced kinetics is thus a “byproduct” of the thermodynamical restructuring. Based on the results, the best Pt catalyst for HER is predicted to be ultrasmall Pt particles without core atoms, a size below ∼20 atoms.

## Abstract 2

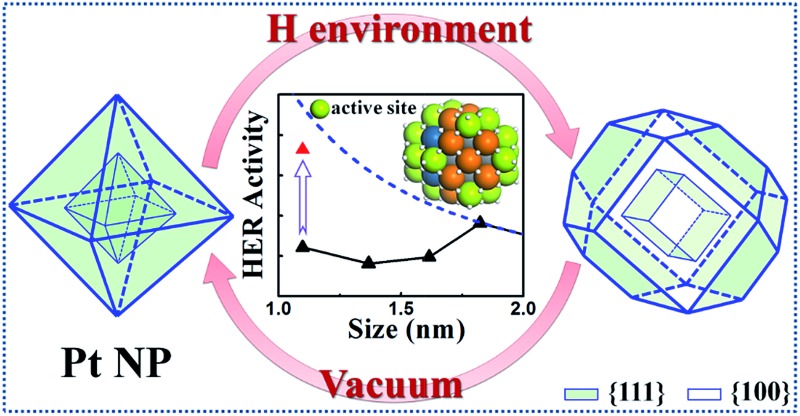
Dynamic catalyst structuring and the hydrogen evolution activity enhancement at nanoscale, as predicted by a first principles global optimization method.

## Introduction

Nanoparticles are common forms or carriers of heterogeneous catalysts^1–3^ and also of wide application in many other fields, *e.g.* as biomedical drug delivery agents^4^ and for energy conversion and storage devices.^5^ Special physicochemical properties emerged at the nanoscale adding a new complexity in understanding and optimizing reactions on nanoparticles. Compared to chunky crystalline materials, nanoparticles are more flexible in morphology and under reaction conditions, the reshaped nanocatalyst may exhibit a completely different activity, either poisoned or promoted after a so-called induction period.^6–9^ Despite the vast amount of research on nanoparticle synthesis and morphology control, major gaps in our knowledge still exist, especially with regard to our molecular level understanding on the *in situ* dynamic restructuring of nanoparticles: this is reflected in our inability to predict whether such restructuring is beneficial or detrimental to catalyst activity. Controlling the nanostructure dynamics for the desirable property, *e.g.* catalytic activity, is paramount for rational catalyst design and is a general goal in nanomaterial applications.^10,11^

Pt is a unique metal with high catalytic performance for a wide range of reactions, and it is perhaps the most efficient HER catalyst in electro- and photocatalytic water splitting.^12,13^ It has been constantly pursued in research to reduce the Pt usage by identifying the optimum particle size for activity. On model single crystalline surfaces, it was however found that HER is only weakly dependent on the crystal facet: the ridged Pt(110) is about two times more active than the (111) and (100) terraces.^14–17^ On going to the nanoscale, there is no consensus on the particle size effect.^18–21^ The presence of the particle-support interaction further complexes the understanding of the particle size effect on activity.^22^ A very recent study by Schweinberger *et al.* using size-selected Pt nanoparticles supported on CdS nanorod shows that the particle of a critical particle size ∼46 atoms (1 nm) can achieve the maximum H_2_ production, whilst the mass activity is the highest when the particle size shrinks down to the subnanoscale with only 8 atoms (Pt_8_).^23^ To date, there is much uncertainty on the physical origin of the HER activity on small nanoparticles. The nature of the active site and the dynamic structure evolution are two key issues that need to be resolved first.

Here we present the first quantum mechanics simulation on the structure dynamics of Pt nanoparticles during HER and quantify its catalytic consequence. The Pt nanoparticle considered in this work is represented by a Pt cluster of ∼1 nm diameter, Pt_44_, which is identified as a magic number size with *O*_h_ symmetry. Significant restructuring-induced promotion is revealed on the Pt_44_ nanoparticle at the HER condition, and theory further predicts that such a promotional effect due to restructuring is prominent only for nanoparticles below ∼1.8 nm. In general, the restructuring as driven by the exothermicity of the adsorption of reaction intermediates may or may not increase the active site concentration that depends on the nature of the reaction and also the particle size.

As both nanoparticle restructuring and catalytic reactions are rare events with high barriers, it presents a challenge to computer simulation since the long simulation times of molecular dynamics, or even the use of enhanced sampling techniques, may not be able to capture the desired reaction patterns. For example, in HER on Pt(111), the barrier of H–H coupling to form H_2_ can be as high as 0.92 eV at the working conditions.^24^ The approach we adopt here is to use the first principles density functional theory (DFT) based stochastic surface walking (SSW) global optimization method,^25–27^ SSW-DFT, to explore the Pt nanoparticle morphology at the HER condition. The recently-developed SSW method is able to visit the minima on PES by following likely pathways, and therefore is a powerful tool for both structure prediction and pathway search.^25^ Using the new technique, we are able to stepwise follow the particle restructuring in a H_2_ atmosphere and determine the HER activity.

## Calculation methods

### DFT calculation

All SSW calculations and the reaction modelling were performed in combination with the DFT calculations as implemented in the SIESTA package^28,29^ with Troullier–Martins norm conserving pesudopotentials.^30^ The exchange–correlation functional utilized was at the generalized gradient approximation level, known as GGA-PBE.^31^ The optimized double-*ζ* plus polarization (DZP) basis set with extra diffuse function was employed for metal. The orbital-confining cut-off was determined from an energy shift of 0.010 eV. The energy cut-off for the real space grid used to represent the density was set at 150 Ry. The Quasi-Newton l-BFGS method is used for geometry relaxation until the maximal force on each degree of freedom is less than 0.01 eV Å^–1^. To correct the zero-point energy for the reaction barrier, the vibrational frequency calculations were performed *via* the finite-difference approach. Transition states (TSs) of the catalytic reaction were searched using the Constrained-Broyden-based TS-searching methods.^32,33^

For all the Pt clusters from Pt_12_ to Pt_46_ (see Fig. S1†), at least four lowest-lying isomers obtained from the SSW-DFT/SIESTA search were further checked using the spin-polarized plane wave calculations with ultrasoft pseudo-potentials^34^ or projected augmented wave^35,36^ pseudo-potentials, as implemented in VASP.^37^ The plane-wave kinetic energy cut-off of 400 eV was used and the exchange–correlation functional utilized was at the generalized gradient approximation level, GGA-PW91 (ref. 38) and GGA-PBE.^31^ Although small Pt clusters in the gas phase are generally spin polarized, the energy contribution of spin polarization is diminished for clusters above 39 atoms (< 0.03 eV).

### SSW calculation

In all SSW simulation, the key parameters utilized are the same with those utilized previously for exploring the PES of carbon and boron clusters,^25–27^ *i.e.* the Gaussian width being 0.6 Å, the number of Gaussian potential being 10.

To identify the global minimum structure of Pt_*x*_ (*x* = 12 to 46) (see Fig. S1†), we set the temperature utilized in Metropolis Monte Carlo as being 3000–5000 K. The higher temperature is used to verify the obtained global minimum structure. In the SSW search, we performed four to ten parallel runs and up to 300 minima are collected at the first stage, from which the most stable configuration is obtained. Next, we verified the result from the most stable configuration of the first stage and collected another 300 minima. This process was repeated until no more further stable configurations were identified at the stage of verification.

For the grand canonical Monte Carlo (GCMC) simulation for Pt_44_H_*x*_ system, the basic procedure of the SSW simulation at each fixed H concentration was the same as that described above for pure Pt clusters. In the GCMC simulation, the major difference was that every 300 SSW steps, we evaluated the chemical potential of adsorbed H atom with respect to that of H in the gas phase Δ*G*_H_ (see below in eqn (2)) based on the current most stable configuration. According to the value Δ*G*_H_ < or >0, we were able decide to accept or refuse the current most stable configuration. To speed up the structure search for reaching the Δ*G*_H_ = 0 equilibrium, the newly-arrived H atoms will be always added to the vacant surface sites, *e.g.* vacant bridge site; the removal of H atoms will always choose the atop H atoms or subsurface H atoms, if present, which are calculated to have the poorest adsorption energy.

### The exothermicity of restructuring under HER condition

In HER, the nanoparticle is in the H_2_ atmosphere and an equilibrium of H chemical potential needs to be achieved at the steady state. The exothermicity of the H adsorption on the bare nanoparticle provides the driving force of the restructuring. This is measured by Δ*G* per Pt atom with reference to Pt_44_ octahedron and H_2_ gas (the standard condition is utilized here), as shown in eqn (1).

1 Δ*G* = [*G*(Pt_44_H_*x*_) – *G*(Pt_44_) – *x*/2*G*(H_2_)]/44

Here *G*(Pt_44_H_*x*_) and *G*(Pt_44_) can be computed from DFT directly by including the zero point energy (ZPE) correction, and *G*(H_2_) is the free energy of the gas phase H_2_ that can be obtained from standard thermodynamics data.^39^

## Results and discussion

### Pt_44_ octahedron

**Fig. 1:**
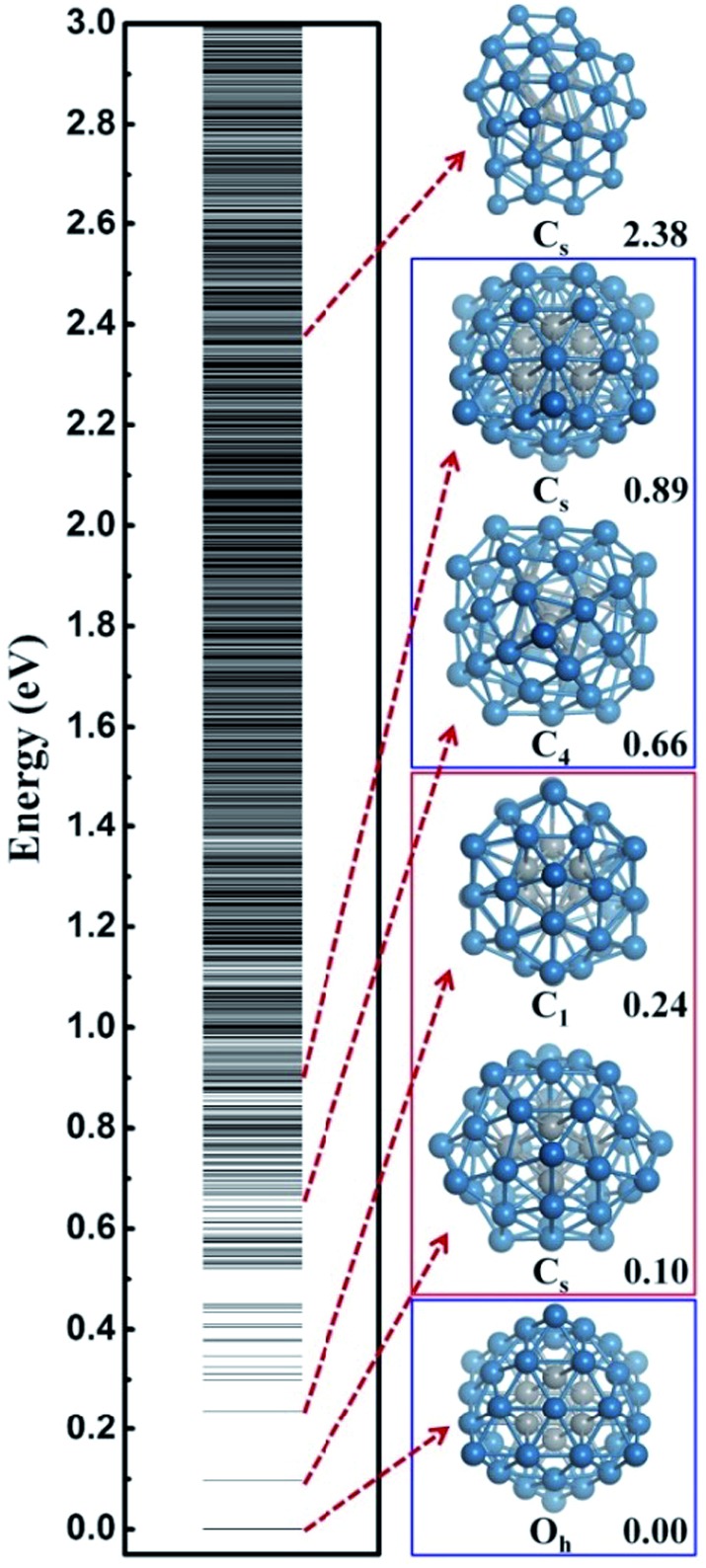
The configurational spectrum of Pt_44_ obtained using SSW-DFT global structure search. Deep blue: shell Pt atoms Grey: core Pt atoms. The PES of Pt_44_ exhibits two distinct energy funnels, a larger funnel represented by the second lowest minimum (SLM), featuring with pentastar structure units on the surface, and a smaller funnel represented by the octahedron GM with a characteristic C_4_ axis. All the stable structures within 0.66 eV above GM are geometrically similar to the SLM. For the GM funnel, the next stable structure appears at 0.66 eV, which can be generated from the GM by rotating half of the shell Pt atoms around the C_4_ axis.

In this work, we utilize Pt_44_ as the model catalyst for investigating the HER on ∼1 nm Pt nanoparticles. Pt_44_ is predicted to a magic number size based on the unbiased SSW-DFT global structure search (see ESI discussion and Fig. S1†), which was also suggested previously^40^ by comparing with other putative high symmetry structures of Pt_44_. From the SSW trajectories 4788 minima of Pt_44_ were collected and the GM of Pt_44_ is found to be a *O*_h_ symmetry octahedron with bulk-like face-centered cubic (fcc) packing, exposing only {111} facets and containing six core atoms and 38 shell atoms, see Fig. 1. It should be mentioned that Pt_44_ is the smallest octahedron of Pt nanoparticle with high stability, which exhibits a remarkable structure similarity to the bulk Pt crystal: the average Pt–Pt distances of Pt_44_ is 2.74 Å, being only 2.7% shorter than that in Pt bulk. This affords Pt_44_ to be a good model for understanding nanoparticle behaviour under reaction conditions.

From all the minima of Pt_44_ collected from SSW trajectories, we have constructed the configurational spectrum of Pt_44_ in Fig. 1 to provide insights into the PES of Pt nanoparticle in general. The structures of typical less stable minima are also shown. In general, the conformation of the Pt nanoparticle is discrete at the energy window 0–0.5 eV above GM and becomes continuous-like above 0.5 eV. Most of the low lying structures have a common core–shell feature as the GM with 6 core atoms and 38 shell atoms. The continuous energy spectrum appears just 0.5 eV above GM, indicating that the Pt nanoparticle is highly mobile and the reconstruction of the shell is kinetically allowed even at ambient conditions.

### Structure evolution under HER condition

**Fig. 2:**
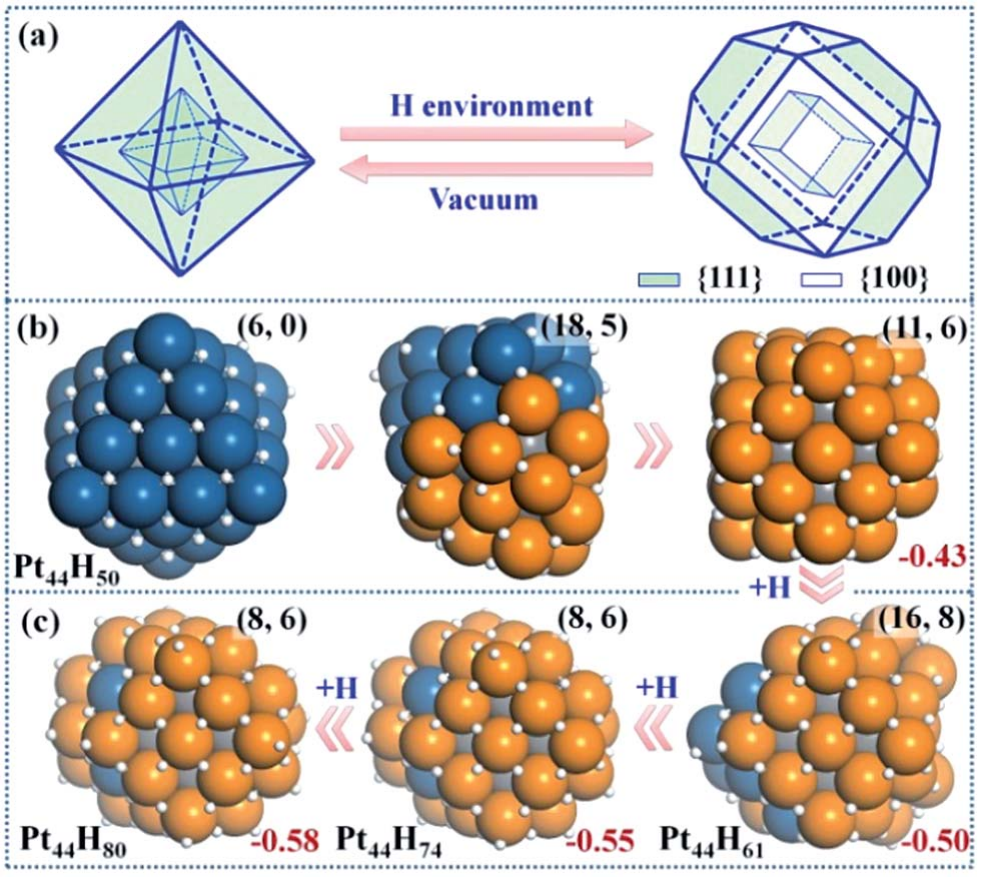
Structure Evolution of Pt_44_ under HER conditions. (a) A polyhedron representation showing the nanoparticle structure evolution from octahedron to tetradecahedron under the HER conditions. (b) The representative structures at Pt_44_H_50_ highlighting the initial stages of {111} to {100} reconstruction; (c) the GM structures at Pt_44_H_61_, Pt_44_H_74_ and Pt_44_H_80_. In the structures, the exothermicity per Pt atom due to the adsorption of H atoms (eqn (1)) is indicated at the right-bottom corner and the numbers of {111} and {100} microfacets are indicated inside the parenthesis of the right-top corner. All the apex Pt in Pt_44_H_80_ are coordinated with five H atoms with one atop H (the H atoms prefer the *fcc* hollow sites on {111} and the bridge sites on {100} terraces). Deep blue: shell Pt atoms associated with {111} facets only; orange: shell Pt atoms associated with at least one {100} facet; grey: core Pt atoms and white: H atoms.

To simulate the structure evolution dynamics at the HER conditions, we consider the Pt_44_/H_2_ system as a grand canonical ensemble where the chemical potential of the adsorbed H atoms will eventually reach equilibrium with that in the gas phase, *i.e.* Δ*G*_H_ → 0. Δ*G*_H_ can be calculated as follows,

2 Δ*G*_H_ = Δ*E*_DFT_ + ZPE – 1/2*G*(H_2_)

where Δ*E*_DFT_ and ZPE are the differential adsorption energy and the zero point energy of the newly-arrived H atom on particle; and *G*(H_2_) is the free energy of H_2_ in the gas phase at the standard state. The grand canonical Monte Carlo (GCMC) simulations based on SSW/DFT global structure search (GCMC/SSW-DFT) are thus performed to investigate the structure evolution. This is obtained by adding/subtracting H atoms into/from the system, Pt_44_H_*x*_, every few hundred (>300) SSW steps of structure search at a fixed H concentration. In fact, at the initial stage of simulation, the addition of H atoms is always energetically preferable as new vacant sites emerge continuously due to the surface reconstruction where newly-arrived H atoms can adsorb. The simulation reaches equilibrium at the stage of Pt_44_H_80_, when Δ*G*_H_ of newly-arrived H atoms become positive. From the trajectories of GCMC simulation, we selected representative structures at several Pt_44_H_*x*_ stages, as shown in Fig. 2, and they are described as follows.

### Pt_44_H_50_

Our GCMC/SSW-DFT simulation starts from a Pt_44_H_50_ octahedron, when {111} facets are fully occupied by H atoms (>1 monolayer, ML) and Δ*G*_H_ starts to exhibit an appreciable decrease due to the switch of the adsorption site for H atom. The simulation shows that the particle leaves the octahedron shape immediately after only 3 SSW steps. The initial reconstruction starts by the collective migration of the vertex Pt atoms of the octahedron, thereby exposing {100} facets (see ESI animation-1†). This is simply because {100} facet is able to adsorb more H atoms than {111} and thus is thermodynamically preferred. In the restructuring, the surface atoms including H diffuse around to find the energetically favorable position and as a result, {100} facets emerge by breaking large {111} facets into small {111} microfacets (Fig. 2b). The surface becomes rough. For Pt_44_H_50_, additional SSW runs were performed to understand the restructuring dynamics (see ESI animation-2†). After a long simulation of 2021 SSW steps, Pt_44_H_50_ becomes significantly different from the initial octahedron, possessing 11 {111} and 6 {100} facets (Fig. 2b), although the core–shell structure still remains: Pt_44_H_50_ has 7 core atoms and 37 shell atoms.

### Pt_44_H_61–80_

With the increase of H coverage, the small {100} microfacets start to merge with each other to yield large {100} facets, and simultaneously the area of {111} facets shrinks. The polyhedron shapes start to reappear as the stable forms and Δ*G*_H_ gradually approaches to zero. The identified GM of Pt_44_H_80_ is found to be a *C*_2h_ tetradecahedron of *fcc* packing, with 8 {111} facets, 6 {100} facets and 18 apex Pt atoms (five or six coordinated Pt atoms shared by at least three facets). Δ*G*_H_ at Pt_44_H_80_ is –0.07 eV.

The GCMC/SSW-DFT simulation conveys two important messages for the HER-driven nanoparticle restructuring: (i) adsorbed H atoms are always more stable on the surface even when the equilibrium coverage is above 2 ML. Importantly, the subsurface H atoms inside the Pt nanoparticle is found to be unstable (see ESI Fig. S2†) since the stronger Pt–Pt bond is energetically preferred compared to the Pt–H bond in forming the particle core. This implies a high stability of Pt nanoparticles under HER conditions. (ii) The restructuring is driven to maximally expose {100} while the core–shell structure of Pt nanoparticles is always kept to minimize the total energy. Only {100} and {111} facets are present at the GM of Pt_44_H_80_. Overall, the core Pt atoms increase from 6 to 8 and the shell Pt atoms decrease from 38 to 36 (the surface density drops) after the restructuring, which is consistent with the typical surface reconstruction observed in surface science studies.^41^

### HER activity

We are now at the position to investigate the HER on the Pt_44_H_80_ polyhedron. We have considered all the likely reaction patterns for the hydrogen evolution *via* the coupling of two adsorbed H atoms, H + H → H_2_, the so-called Tafel mechanism in electrocatalytic HER that is preferable at high H coverage conditions (see our recent work on HER kinetics on surfaces^24^ where the electrochemical potential and solvation effect have been considered; here we follow the main conclusions obtained there).

**Fig. 3:**
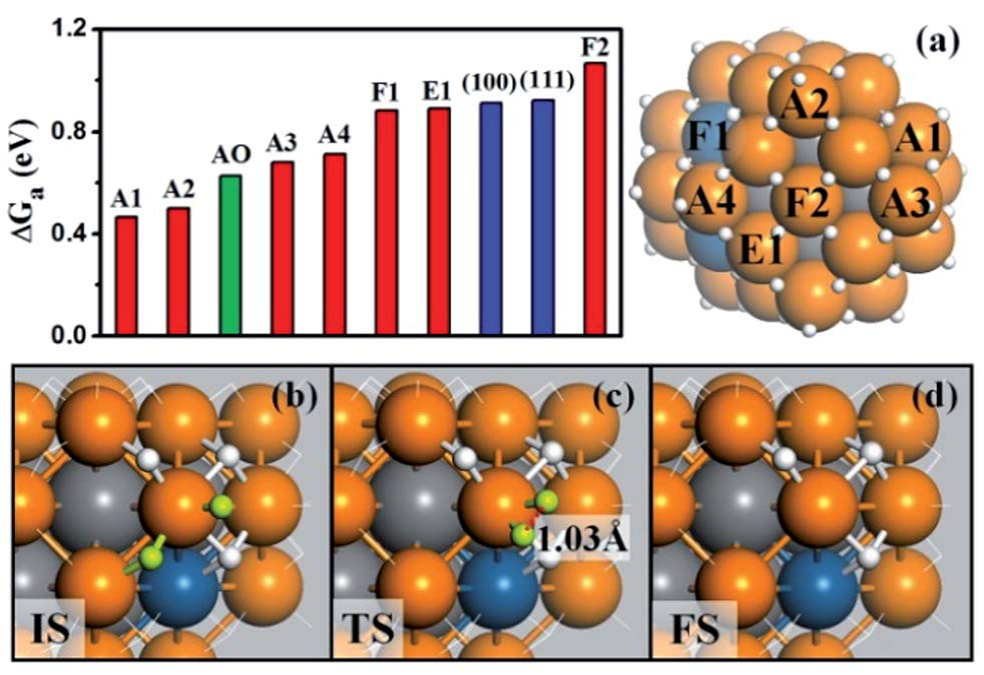
(a) The free energy barriers (Δ*G*_a_) of H–H coupling on Pt_44_H_80_. The sites are as indicated on the right-hand particle. Among 18 apex sites, there are ten A1, four A2, two A3 and two A4 sites. Also shown are Δ*G*_a_ on the extended (111), (100) surfaces and the apex sites of the unreconstructed Pt_44_H_48_ (AO); (b–d) the reaction snapshots for the lowest barrier reaction channel at the apex A1 site. The reaction features with the atop H reacting with a neighboring bridging H (both H highlighted by yellow color), where the apex Pt atom is coordinated with five H atoms. The color scheme is as in Fig. 2.

Fig. 3a shows that the calculated free energy barriers (Δ*G*_a_) of H–H coupling on Pt_44_H_80_ span from 0.47 to 1.07 eV depending on the local sites. Unexpectedly, Δ*G*_a_ at the apex sites are 0.47–0.71 eV, which is much lower than that on the {111}, edge and {100} sites, 0.88, 0.88 and 1.07 eV, respectively. We also noticed that the calculated Δ*G*_a_ of the H–H coupling on the {111} facets of Pt_44_H_80_ (F1) is in fact similar to that on the extended Pt(111) (∼0.9 eV). The optimized structures of the transition state (TS) are also similar in two cases (see ESI Fig. S3†). Similarly, Δ*G*_a_ at the apex sites of the unreconstructed Pt_44_H_48_ octahedron (AO) is also in the Δ*G*_a_ range of the apex sites on Pt_44_H_80_. These results indicate that the HER activity can be assessed largely by the local geometry of the Pt site.

**Fig. 4:**
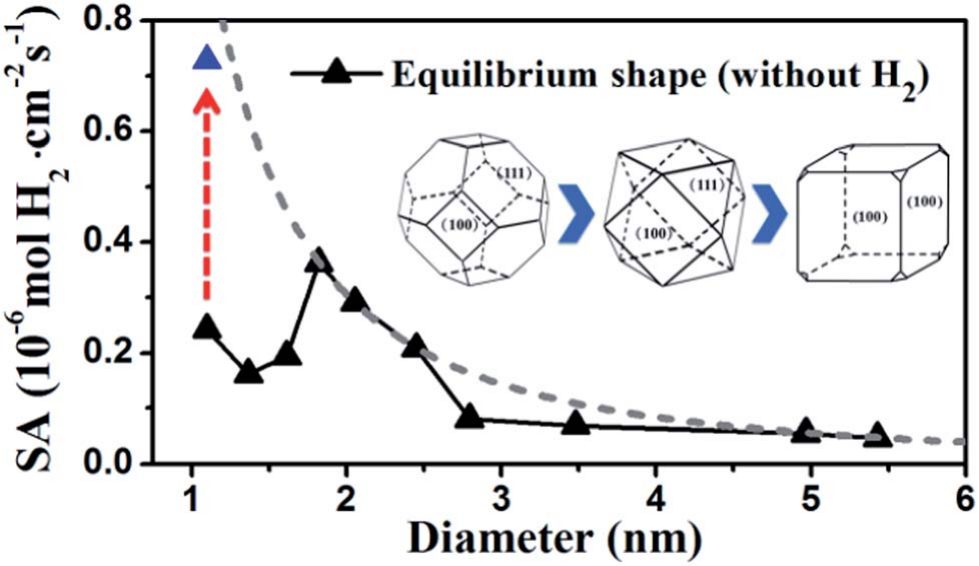
Estimated HER activity (using eqn (3)) of Pt nanoparticles with the equilibrium shape in solution, *i.e.* no restructuring due to H_2_, (black curve) and with only the {100}-dominated truncated cubic shape (grey dash curve). The nanoparticle equilibrium shape in solution is taken from experiment^42^ and our recent work,^43^ which exhibits a gradual transition from octahedron at very small particles to truncated octahedron, to cuboctahedron and to the {100}-dominated truncated cube at large particles. The grey dash curve represents the activity limit after the restructuring.

By identifying the critical role of apex sites and the local reactivity in HER, we can discuss their implication to HER catalysis. In Fig. 4, we first estimated the HER activity of Pt particles on differently sized as-synthesized nanoparticles at the equilibrium shape in solution, *i.e.* no restructuring due to H_2_ (data taken from experiment and our recent study).^42,43^ We then count the apex, {111} and {100} and edge sites of the particles and sum the overall HER rate (specific activity of HER, SA, mol cm^–2^ s^–1^) on all the sites based on microkinetics, as shown in eqn (3).3
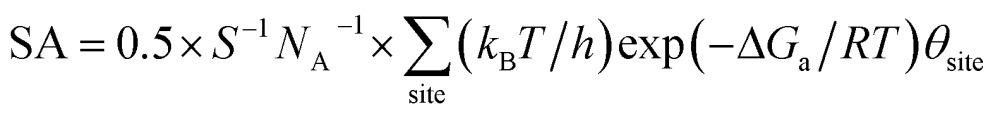
where *k*_B_ is Boltzmann's constant and *h* is Planck's constant (*k*_B_ *T*/*h* is 6.25 × 10^12^ at 298.15 K from classic TS theory); Δ*G*_a_ is the estimated free energy barrier statistically averaged according to the data in Fig. 3a, *i.e.* 0.48 eV for apex sites, 0.88 eV for terrace sites and 0.89 eV for edge sites; *θ*_site_ is the number of the active sites on the nanoparticles; *S* is the surface area. Here we assume the same HER activity at the same type of site based on the fact of local reactivity of HER identified above. The calculated HER rate on the nanoparticle is thus plotted in Fig. 4 black curve, showing the maximum activity around ∼1.8 nm when the highest concentration of the apex site (per unit surface area) is reached. It should be mentioned that the HER activity estimated in the black curve agrees reasonably with the kinetic data reported in experiment, validating largely the local reactivity assumption. For example, Hoshi *et al.* reported 1.21 mA cm^–2^ for electrocatalytic HER on ∼3 nm Pt nanoparticles that corresponds to ∼6.3 × 10^–9^ mol cm^–2^ s^–1^ with the apparent Δ*G*_a_ of 0.54 eV (eqn (3)), while the estimated Δ*G*_a_ utilized in Fig. 4 is 0.48 eV and the difference in the rate is no more than 10 times.

Furthermore, we may also consider the situation after the nanoparticle restructuring at the HER condition. Although we do not know the exact atomic structure, this work does show that {100} is the direction of restructuring and thus the {100}-dominated truncated cube would be the preferred shape starting from small particles, where the apex sites can reach 24 per particle. We therefore estimate the HER rate as a possible maximum limit due to restructuring using the same approach above, as plotted in the grey dash curve in Fig. 4. Indeed, the trend for the large increase of HER activity of Pt_44_ after restructuring is correctly reflected in the figure (the red arrow). Interestingly, Fig. 4 predicts that for large nanoparticles above 1.8 nm, the restructuring of nanoparticles, although should occur as well, does not enhance the HER activity appreciably. The activity decays very slowly above 3 nm, when the activity can be regarded as insensitive to the particle size.

By contrast, for very small nanoparticles (*e.g.* 1 nm), the activity can be dramatically higher, which is caused by the dynamic restructuring at the HER condition that creates a high concentration of five or six coordinated apex sites per surface area. Along this line, we expect that ultrasmall Pt clusters without core atoms have the highest HER activity because all Pt atoms are on the surface with low coordination, where the concentration of apex sites can be maximized at the HER condition. This corresponds to a size of less than ∼20 atoms (see ref. 40 and also ESI Fig. S1†), which may rationalize the highest photocatalytic HER activity of Pt_8_ observed recently.^23^

## Conclusions

New DFT-based global optimization theoretical methods allow the observation of the dynamic catalyst structure evolution and the quantification of the activity change of Pt nanoparticles for HER. Unexpectedly, we found that HER occurs preferentially on Pt low-coordinated apex Pt sites, which totally dominates the activity for Pt nanoparticles. The restructuring of nanoparticles can promote HER, but appreciably only below a certain size threshold, ∼2 nm, where the apex sites dynamically created can reach the maximum concentration. The subnano Pt clusters without core atoms are predicted to have the highest HER activity.
