# Supplementary figures and images for: Restructuring and Hydrogen Evolution on Pt Nanoparticle
Source: Chem Sci. 2014 Nov 26;6(2):1485–90. doi: 10.1039/c4sc02806f (PMC5811100; doi:10.1039/c4sc02806f)

## Slide 1
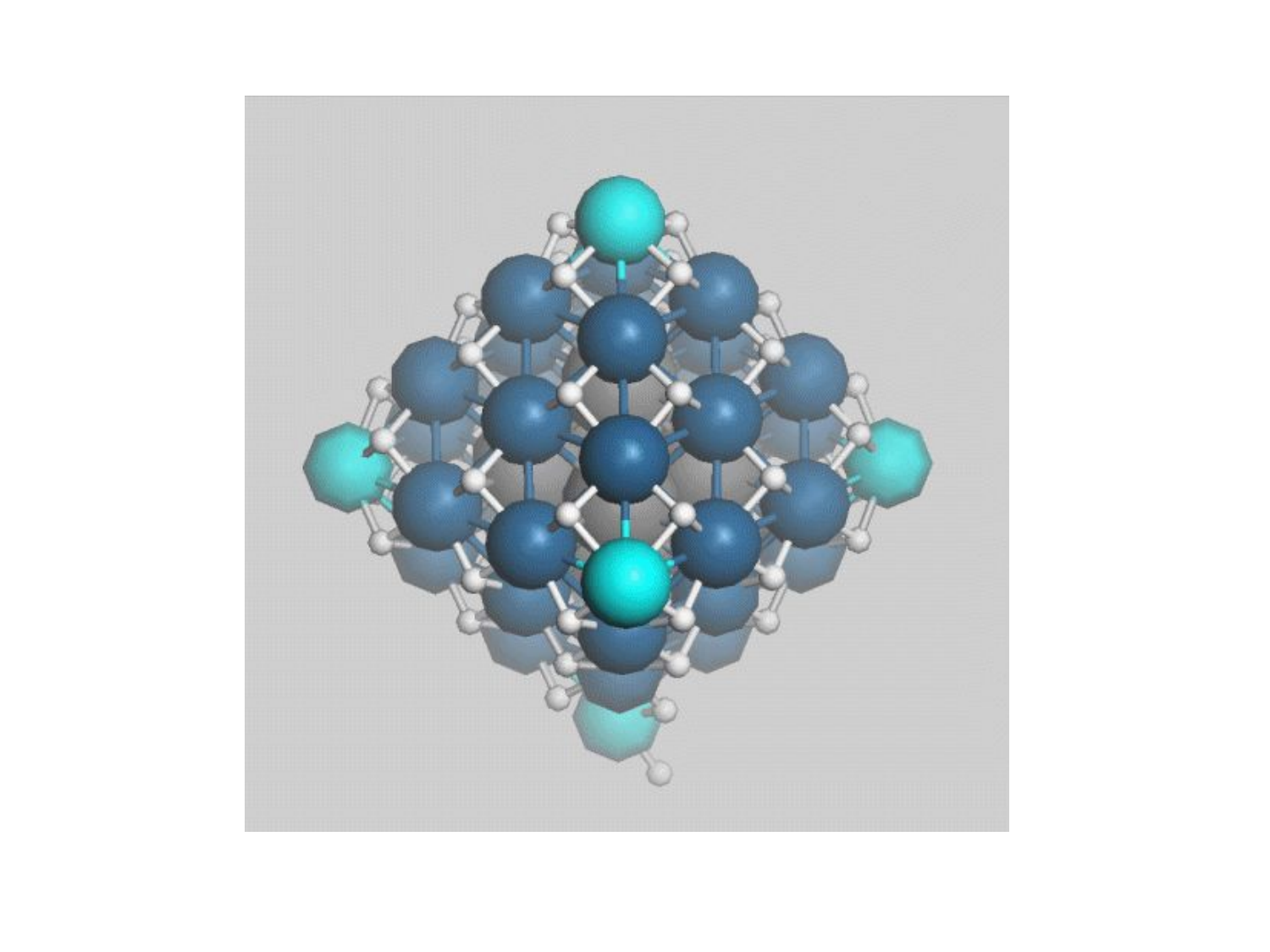

Supplement: Supplementary file 1 [file SC-006-C4SC02806F-s001.zip › animation-1.pptx]

## Slide 1
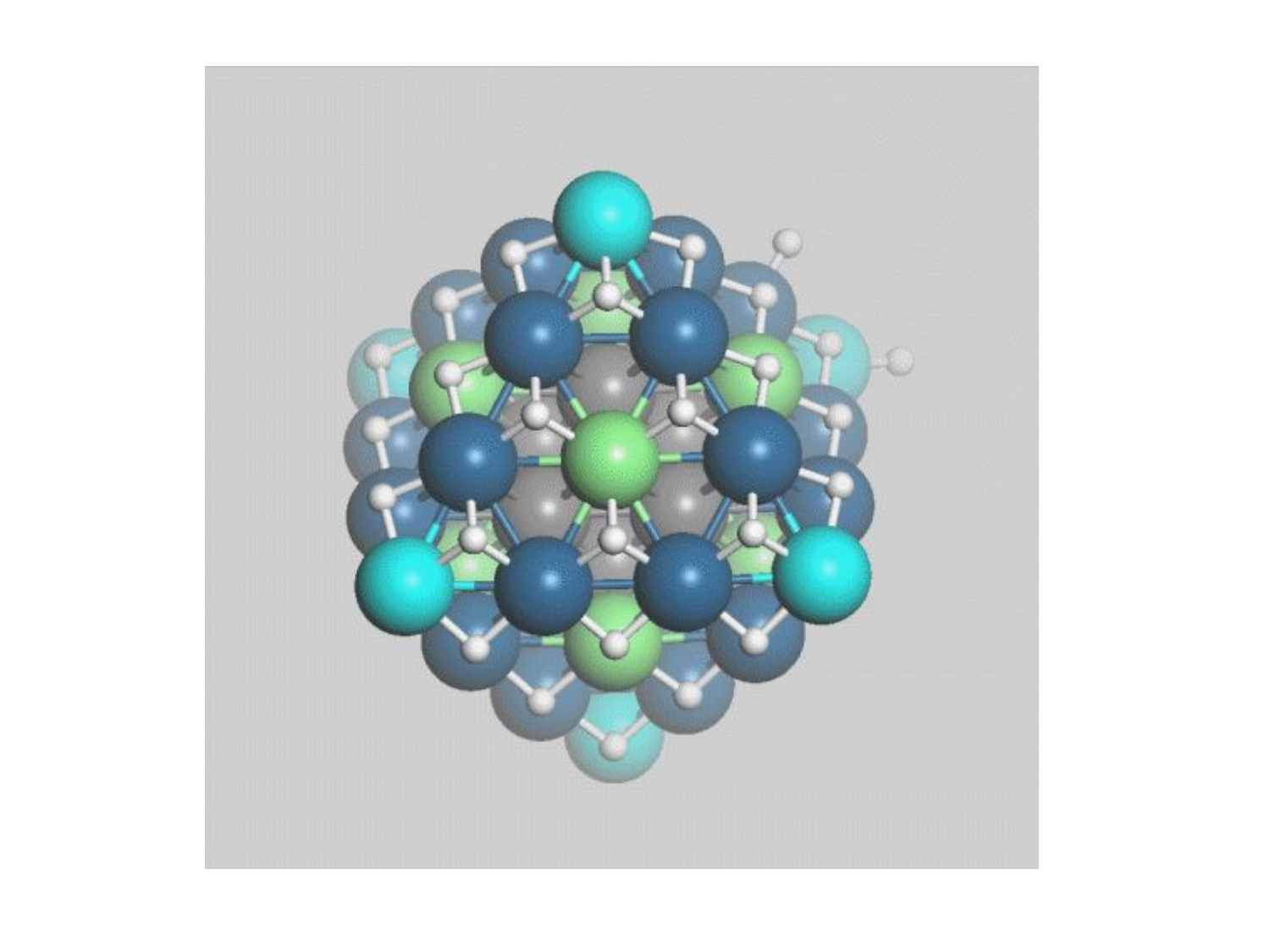

Supplement: Supplementary file 1 [file SC-006-C4SC02806F-s001.zip › animation-2.pptx]
