# Supplementary material for: Restructuring and Hydrogen Evolution on Pt Nanoparticle
Source: Chem Sci. 2014 Nov 26;6(2):1485–90. doi: 10.1039/c4sc02806f (PMC5811100; doi:10.1039/c4sc02806f)
Supplement: Supplementary file 2 [file SC-006-C4SC02806F-s002.pdf]

Electronic Supplementary Material (ESI) for Chemical Science.
This journal is © The Royal Society of Chemistry 2014

Supplementary information

Restructuring and Hydrogen Evolution on Pt Nanoparticle

Guang-Feng Wei, Zhi-Pan Liu*

Shanghai Key Laboratory of Molecular Catalysis and Innovative Materials, Department of Chemistry, Key Laboratory of Computational Physical Science (Ministry of Education), Fudan University, Shanghai 200433, China

Contents

1. **On the structures of Pt clusters**

2. **The stability of the subsurface H atoms in Pt cluster**

4. **HER on {111} facets of $Pt_{44}H_{80}$**

5. **The XYZ coordinates for the GM of Pt_{44} and $Pt_{44}H_{80}$**

1. On the structures of Pt clusters

Nano and subnano Pt clusters have been extensively studied for their relevance to catalysis (e.g. interaction with molecules)[16-18]. However, the (most stable) structure of small Pt clusters is generally highly controversial due to the complexity of the potential energy surface of transition metal clusters. A famous example is Pt_{55} cluster, which was suggested be a magic cluster with I_h symmetry, the same as Lennard-Jones particles. However, Apra et al.[19] found that Pt_{55} might be amorphous like since the I_h structure is much less stable. Li et al.[20] shows that in fact, 55 atom clusters of many transition metals is less stable than its neighboring 54 or 56 clusters.

In this work, we aim to identify a well-defined structure as a starting point for studying HER and thus we have searched for the magic number size of (sub)nano Pt particles based on the SSW-DFT global structure search. Since the structure of many of these small Pt clusters have been analyzed in the previous work[21-22], one purpose here is also to confirm the magic number size reported. Indeed, we found that the global minimum (GM) of Pt_{44} is a O_h structure (Fig. S1b) and it is the magic number of ~ 1 nm with higher relative stability compared to the neighboring clusters. To make this clearer, we also show the GM structures of Pt_N (N= 41~46), nearby the magic cluster Pt_{44}, in Fig. S1a. The GM structure of Pt_{42}, Pt_{43} and the second lowest minima of Pt_{40} and Pt_{45} are similar with the O_h Pt_{44}, which can be generated by adding or removing the apex Pt atoms from Pt_{44}. For Pt_{41}, Pt_{45} and Pt_{46}, the low symmetry structures turn out to be more stable and become the GM structure (they are not magic numbers).

For the other sizes investigated (as shown in Fig. S1), we found that the magic number size appears at Pt_{14}, Pt_{18}, Pt_{22}, Pt_{27}, Pt_{36}, being in general consistent with those suggested previously[21], although the exact GM structure from this work can be different. This is mainly due to additional new structure being identified using SSW-DFT global search, which turns out to be more stable than the previously reported GM. For example, the GM structure identified by our SSW-DFT global search of Pt_{36} is a newly found D_{3h} triangular prism, which contains 3 core atoms and 33 surface atoms. This structure is 1.51 eV more stable than the previously reported 4×9 (four layers and nine atoms per layer) cuboid structure[21].

Here we discuss briefly on the magic cluster size of the small Pt clusters (which is not the focus in this work and will be followed up in our future work). For example, Kumer and Kawazoe[21] reported that the cube isomer with simple cubic packing has the lowest energy for Pt_{25}~Pt_{38} and the fcc packing O_h structures becomes more stable for the larger clusters. According to our results, the GM of the magic cluster of Pt_{27} is indeed the same simple cubic structure, but such cube structures are only stable in a small range (from Pt_{25} to Pt_{28}). For all Pt_N clusters (N = 12~46) studied in this work, only 5 cluster sizes (N = 14, 40, 42, 43, 44) are identified to have the the fcc-packing GM structure. For many non-magic size clusters, the GM structure is in fact amorphous like, similar to Pt_{55} mentioned above. A nice example is Pt_{38}, a low symmetry (C_1) structure is identified to be GM, which is 1.92 eV and 1.75 eV more stable than the cube C_{2v} structure and truncated octahedron O_h structure (the most stable structure of LJ cluster).

It might also be mentioned that the GM structures of Pt clusters below Pt_{22} have no core atoms with all Pt atoms being on the surface. The core-shell structure starts from Pt_{23}, where one core

atom is present. The number of core atoms then increases gradually: there are six core atoms at Pt_{44} octahedron.

Magic number size in Pt_N clusters (N=12 to 46) from SSW-DFT search

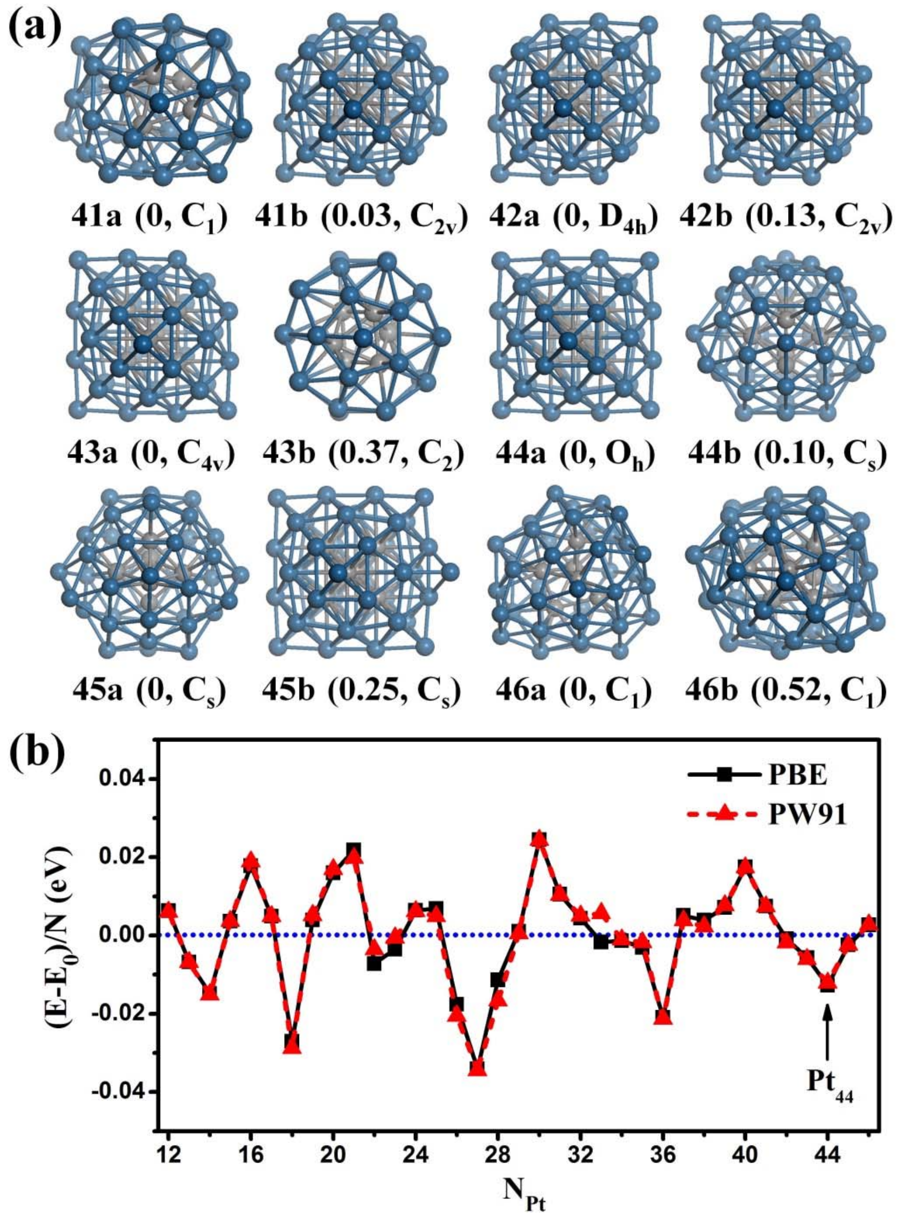

Fig. S1 (a) Low lying isomers of Pt_N (N= 41~46) clusters. For one particular size N, Na is the GM and Nb is the second lowest minimum. The energy relative to the GM and the symmetry are listed in the brackets. Deep blue: Shell Pt atoms; Grey: core Pt atoms. (b) The relative energies of Pt_N (N=12~46) clusters calculated with two different DFT functionals, PBE and PW91. According to the fitted relation between E and N, we arrive at $E_0 = 2.13431 – 5.61731 N^{1/3} + 1.58801 N^{2/3} – 0.162408 N$ for PBE, and $E_0 = 1.93275 –5.39152 N^{1/3} + 1.50598 N^{2/3} – 0.152585 N$ for PW91. Two DFT functionals yield basically the same conclusion for the magic number size for these Pt clusters.

2. The stability of the subsurface H atoms in Pt cluster

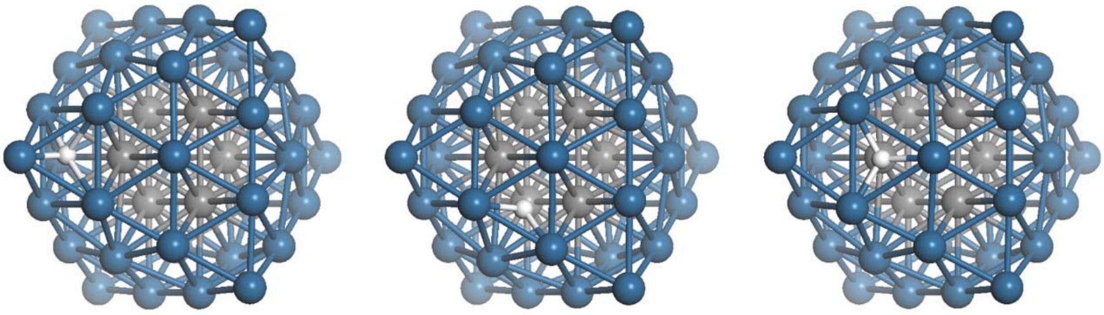

A (+0.93 eV) B (+0.74 eV) C (+0.68 eV)

Fig. S2 Three different structures with one subsurface H in the O_h Pt_{44} cluster and the corresponding adsorption free energy (ΔG_{ad}) of the subsuface H (in brackets). Deep blue: Shell Pt atoms; Grey: core Pt atoms, White: H atoms.

During the SSW-DFT global search of $Pt_{44}H_x$ clusters, we found that the minima contains the subsurface H atoms are generally not stable and will not be retained during the GCMC evolution. No subsurface H atoms are present for the GM of $Pt_{44}H_x$, including $Pt_{44}H_{80}$. This is an very interesting phenomena, indicating the subsurface H is not stable in Pt nanoparticles, which renders a long-term stability of Pt nanoparticle. To provide better understanding into this issue, we have compared the adsorption free energy (ΔG_{ad}) of one subsuface H in the O_h Pt_{44} cluster. The H at three different subsurface sites are considered. As shown in Fig. S2, the value of ΔG_{ad} with respect to the gas phase H_2 (standard state) are all positive for three situations. At the most stable tetrahedral subsurface site (Fig. S2 C),ΔG_{ad} is already +0.68 eV. This result supports the fact that the Pt-Pt interaction is much stronger than the Pt-H interaction, and since the presence of subsurface H must weaken the Pt-Pt interaction, the H does not prefer the subsurface positions in Pt cluster. On the other hand, ΔG_{ad} for the first H on the surface of Pt_{44} cluster is -0.37 eV (at an edge site). Comparing ΔG_{ad} of H on the surface and at the subsurface, the energy difference is more than 1 eV, which suggests that thermodynamically it is not possible for the diffusion of H atoms into Pt cluster.

References:

[1] J. M. Soler, E. Artacho, J. D. Gale, A. Garcia, J. Junquera, P. Ordejon, D. Sanchez-Portal, *J. Phys.-Condes. Matter* **2002**, *14*, 2745-2779.

[2] J. Junquera, O. Paz, D. Sanchez-Portal, E. Artacho, *Physical Review B* **2001**, *64*.

[3] N. Troullier, J. L. Martins, *Physical Review B* **1991**, *43*, 1993-2006.

[4] J. P. Perdew, K. Burke, M. Ernzerhof, *Physical Review Letters* **1996**, *77*, 3865-3868.

[5] H. F. Wang, Z. P. Liu, *J. Am. Chem. Soc.* **2008**, *130*, 10996-11004.

[6] C. Shang, Z. P. Liu, *J. Chem. Theory Comput.* **2010**, *6*, 1136-1144.

[7] G. Kresse, J. Hafner, *J Phys-condens Mater* **1994**, *6*, 8245-8257.

[8] P. E. Blochl, *Phys Rev B* **1994**, *50*, 17953-17979.

[9] G. Kresse, D. Joubert, *Phys Rev B* **1999**, *59*, 1758-1775.

[10] G. Kresse, J. Furthmuller, *Comp Mater Sci* **1996**, *6*, 15-50.

[11] J. P. Perdew, Y. Wang, *Phys Rev B* **1992**, *45*, 13244-13249.

[12] C. Shang, Z. P. Liu, *J. Chem. Theory Comput.* **2013**, *9*, 1838-1845.

[13] X. J. Zhang, C. Shang, Z. P. Liu, *J. Chem. Theory Comput.* **2013**, *9*, 3252-3260.

[14] H. J. Zhai, Y. F. Zhao, W. L. Li, Q. Chen, H. Bai, H. S. Hu, Z. A. Piazza, W. J. Tian, H. G. Lu, Y. B. Wu, Y. W. Mu, G. F. Wei, Z. P. Liu, J. Li, S. D. Li, L. S. Wang, *Nat. Chem.* **2014**, *6*, 727-731.

[15] D. R.LIDE, 84th ed., CRC press, **2003-2004**.

[16] L. Tang, B. Han, K. Persson, C. Friesen, T. He, K. Sieradzki, G. Ceder, *J. Am. Chem. Soc.* **2010**, *132*, 596-600.

[17] L. Li, A. H. Larsen, N. A. Romero, V. A. Morozov, C. Glinsvad, F. Abild-Pedersen, J. Greeley, K. W. Jacobsen, J. K. Norskov, *J. Phys. Chem. Lett.* **2013**, *4*, 222-226.

[18] T. Jacob, *Fuel Cells* **2006**, *6*, 159-181.

[19] E. Apra, F. Baletto, R. Ferrando, A. Fortunelli, *Phys. Rev. Lett.* **2004**, *93*.

[20] S. F. Li, X. J. Zhao, X. S. Xu, Y. F. Gao, Z. Y. Zhang, *Phys. Rev. Lett.* **2013**, *111*.

[21] V. Kumar, Y. Kawazoe, *Physical Review B* **2008**, *77*.

[22] X. L. Wang, D. X. Tian, *Comput. Mater. Sci.* **2009**, *46*, 239-244.

3. HER on {111} facets of $Pt_{44}H_{80}$

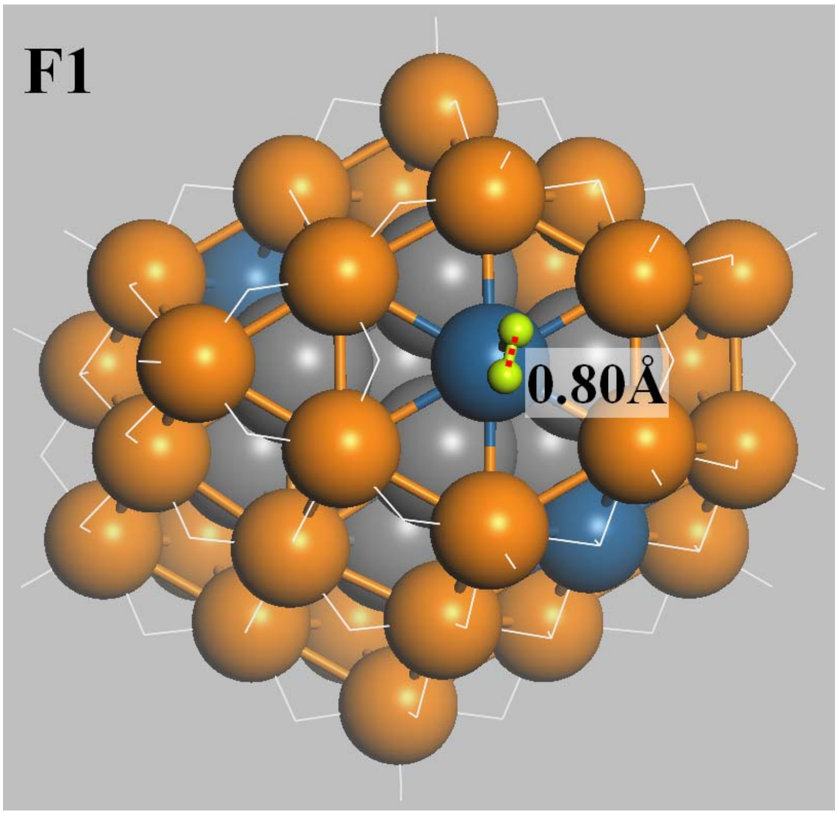

Fig. S3. The optimized structures for the transition state of H-H coupling on {111} facets of $Pt_{44}H_{80}$ (site F1, shown in Figure 3a). The color scheme as those described in Figure 2 and Figure 3.

4. The XYZ coordinates for the GM of Pt_{44} and $Pt_{44}H_{80}$

Pt_{44}:

Pt	2.780918	4.003260	-2.677956
Pt	-2.944262	-4.018862	2.743140
Pt	3.809291	-0.046231	4.086782
Pt	-3.970939	0.026635	-4.022587
Pt	2.795694	-3.949062	-2.762136
Pt	-2.956763	3.932736	2.827626
Pt	0.496794	2.732631	-3.241176
Pt	-0.659565	-2.749763	3.303721
Pt	2.879290	1.333273	-2.797734
Pt	3.236861	2.709065	-0.383344
Pt	0.854319	4.109534	-0.827308
Pt	-3.043892	-1.348476	2.863574
Pt	-3.399570	-2.722685	0.450523
Pt	-1.016342	-4.126280	0.892396
Pt	3.574123	1.379462	1.837221
Pt	3.576681	-1.421274	1.806877
Pt	1.556611	-1.445380	3.745651
Pt	1.553251	1.354358	3.775449
Pt	-3.736434	-1.394467	-1.770091
Pt	-3.739757	1.405161	-1.742930
Pt	-1.719461	1.427340	-3.682087
Pt	-1.715354	-1.373247	-3.709406
Pt	0.505638	-2.678212	-3.296029
Pt	0.867602	-4.102985	-0.914367
Pt	3.244452	-2.702804	-0.441300
Pt	2.884325	-1.277288	-2.825795
Pt	-0.667273	2.660681	3.362091
Pt	-1.028629	4.085893	0.979798
Pt	-3.406101	2.688403	0.504715
Pt	-3.047544	1.261848	2.890941
Pt	1.411926	-3.065190	1.551577
Pt	0.625409	0.031178	-3.623884
Pt	-0.788543	-0.048600	3.687894
Pt	-1.564000	-3.034075	-1.548549
Pt	1.402876	3.019800	1.616591
Pt	-1.572722	3.047400	-1.487722
Pt	-3.760892	-0.018505	0.588061
Pt	3.593203	0.004963	-0.521713
Pt	-1.060359	-1.378836	0.959341
Pt	1.246508	-0.020802	1.418096

Pt	-1.064584	1.338689	0.988166
Pt	-1.410008	0.004980	-1.352863
Pt	0.896390	1.363562	-0.893555
Pt	0.900810	-1.354779	-0.922591

$Pt_{44}H_{80}$:

H	6.190823	-3.889182	0.152327
H	-6.165477	4.180152	-0.017523
H	-2.277212	-3.904047	1.302662
H	3.963031	4.430973	0.100895
H	1.026902	-3.922411	5.265644
H	4.009025	-0.829898	5.383632
H	-4.121130	1.108846	5.303156
H	-4.281723	2.336099	3.178936
H	2.303481	-3.799735	3.456043
H	2.105102	-3.988358	1.201311
H	2.230170	-1.995318	5.146050
H	4.214141	-2.036137	3.260251
H	-0.232522	-3.858626	3.423609
H	-4.302719	-1.983103	1.291184
H	-2.164089	-2.086967	3.328599
H	-0.302489	-2.147154	5.119330
H	-0.054537	0.136860	5.184613
H	-6.253612	-1.918563	0.063404
H	-4.251468	-0.139495	3.243796
H	-2.252842	-3.903989	-1.170413
H	-0.032975	-4.023785	-1.034528
H	-0.141009	-3.862749	-3.275481
H	4.374881	-3.809080	-1.149280

H	4.344265	-3.806442	1.402422
H	2.392798	-3.798535	-3.269907
H	2.125691	-3.985294	-1.021055
H	-0.051977	-4.017856	1.181919
H	0.158870	4.149915	3.430457
H	-2.360883	4.090794	3.404864
H	0.021459	4.296039	1.163263
H	2.226102	4.198488	1.333620
H	0.216719	4.112122	-3.304862
H	-4.362014	4.095085	1.287910
H	-2.307158	4.047945	-3.359686
H	-2.111781	4.249047	-1.087299
H	2.240086	4.189423	-1.155376
H	-4.329817	4.073976	-1.288183
H	0.034929	4.293997	-1.066544
H	-2.124938	4.269781	1.140618
H	-2.366877	-0.124164	5.110859
H	2.268842	0.421649	5.152253
H	0.194564	2.421451	5.135135
H	-2.336372	2.297858	5.108490
H	-0.187263	-2.164101	-4.985695
H	-2.271812	-0.183338	-5.029061
H	2.389774	0.402704	-4.964362
H	0.309618	2.389536	-4.998013
H	-2.225464	2.224684	-5.036894
H	0.059734	0.111996	-5.045261
H	2.354623	-2.000822	-4.954644
H	6.059287	-2.032152	-1.094835
H	6.025690	-2.020536	1.382705
H	6.051585	0.432789	-1.108409
H	6.017392	0.445693	1.382084
H	-	2.311845	-1.251319

	6.002347		
H	-6.066879	-0.087948	1.253905
H	-6.282920	1.131330	3.063940
H	-6.030283	-0.144245	-1.247821
H	-4.205396	2.289082	-3.157904
H	4.303843	-2.049860	-3.023111
H	-6.030673	2.328542	1.248810
H	4.296559	0.431375	-3.056589
H	2.156918	2.348611	-3.194399
H	4.224665	2.339278	-1.111911
H	-4.242546	-2.059482	-1.153139
H	-4.220328	-0.185988	-3.159184
H	-2.095625	-2.103102	-3.235783
H	2.077511	2.371382	3.379549
H	4.200608	2.343761	1.326102
H	4.208160	0.440453	3.295898
H	4.144113	-0.834310	-5.154475
H	6.313004	-0.802082	-2.891554
H	6.232078	-0.785585	3.174631
H	-4.006639	1.058739	-5.274810
H	-6.228460	1.063256	-3.045607
H	-1.116346	4.201285	5.259078
H	-1.026566	4.158398	-5.163096
H	1.148678	-3.921852	-5.100902
H	-4.004915	-4.124253	0.060313
H	6.211041	2.249596	0.125069
Pt	-5.006701	3.082467	0.002507
Pt	2.948499	-0.834499	0.103348
Pt	-5.058725	1.098084	2.039467

Pt	-5.041761	-0.893475	0.033212
Pt	-5.019777	1.062723	-2.003769
Pt	2.996654	1.176574	-1.904268
Pt	2.926025	3.224801	0.097035
Pt	2.945283	1.185480	2.106394
Pt	-2.954254	-2.930282	0.052617
Pt	-0.962154	-2.894552	2.084075
Pt	1.032732	-3.129797	0.081660
Pt	-0.919122	-2.903928	-1.960873
Pt	-1.080276	3.108589	4.091983
Pt	0.926561	1.153815	4.144572
Pt	-1.019413	-0.871186	4.125271
Pt	-3.059493	1.081689	4.108672
Pt	-0.929771	-0.895522	-4.007978
Pt	-2.982168	1.029314	-4.047709
Pt	-0.999446	3.065593	-3.995674
Pt	1.020283	1.126043	-3.984964
Pt	5.019535	1.201097	0.121368
Pt	0.980387	-0.843081	2.058221
Pt	-3.023330	3.113014	2.026090
Pt	1.028589	-0.852715	-1.894424
Pt	0.964538	1.203134	0.078498
Pt	0.904569	3.182501	2.099334
Pt	-0.974525	-0.927177	0.060175
Pt	-2.964686	-0.915242	-1.966849
Pt	-0.985753	1.104027	-1.928462
Pt	-1.039906	3.400886	0.043084
Pt	-	1.112448	0.035245

	2.944978		
Pt	-2.984259	3.081120	-1.981855
Pt	-1.027286	1.127384	2.037899
Pt	0.947709	3.164452	-1.955273
Pt	-3.002359	-0.893892	2.052318
Pt	5.027404	-2.796419	0.132685
Pt	5.077847	-0.797308	-1.880122
Pt	5.022273	-0.785272	2.132961
Pt	2.976684	-0.785444	4.164857
Pt	0.996959	-2.820273	4.106624
Pt	1.096003	-2.823838	-3.939329
Pt	3.084057	-0.795347	-3.958659
Pt	2.986916	-2.822008	2.093066
Pt	3.039236	-2.824858	-1.884168
